# Cesarean delivery and early childhood diseases in Bangladesh: An analysis of Demographic and Health Survey (BDHS) and Multiple Indicator Cluster Survey (MICS)

**DOI:** 10.1371/journal.pone.0242864

**Published:** 2020-12-03

**Authors:** Mohammad Nayeem Hasan, Muhammad Abdul Baker Chowdhury, Jenifar Jahan, Sumyea Jahan, Nasar U. Ahmed, Md Jamal Uddin

**Affiliations:** 1 Department of Statistics, Shahjalal University of Science & Technology, Sylhet, Bangladesh; 2 Department of Emergency Medicine, University of Florida College of Medicine, Gainesville, FL, United States of America; 3 Department of Epidemiology, Florida International University, Miami, FL, United States of America; Anglia Ruskin University, UNITED KINGDOM

## Abstract

**Introduction:**

The rate of cesarean delivery (C-section) has been increasing worldwide, including Bangladesh, and it has a negative impact on the mother and child's health. Our aim was to examine the association between C-section and childhood diseases and to identify the key factors associated with childhood diseases.

**Methods:**

We used four nationally representative data sets from multiple indicator cluster survey (MICS, 2012 and 2019) and Bangladesh Demographic and Health Survey (BDHS, 2011and 2014) and analyzed 25,270 mother-child pairs. We used the frequency of common childhood diseases (fever, short or rapid breaths, cough, blood in stools, and diarrhea) as our outcome variable and C-section as exposure variable. We included mother’s age, place of residence, division, mother’s education, wealth index, child age, child sex, and child size at birth as confounding variables. Negative binomial regression model was used to analyze the data.

**Results:**

In the BDHS data, the prevalence of C-section increased from 17.95% in 2011 to 23.33% in 2014. Also, in MICS, the prevalence almost doubled over an eight-year period (17.74% in 2012 to 35.41% in 2019). We did not observe any significant effect of C-section on childhood diseases in both surveys. Only in 2014 BDHS, we found that C-section increases the risk of childhood disease by 5% [Risk Ratio (RR): 1.05, 95% CI: 0.95, 1.17, p = 0.33]. However, the risk of childhood disease differed significantly in all survey years by division, child's age, and child’s size at birth after adjusting for important confounding variables. For example, children living in Chittagong division had a higher risk [(2011 BDHS RR: 1.22, 95% CI: 1.08, 1.38) and (2019 MICS RR: 1.21, 95% CI: 1.08, 1.35)] of having disease compared to Dhaka division. Maternal age, education, and wealth status showed significant differences with the outcome in some survey years.

**Conclusion:**

Our study shows that C-section in Bangladesh continued to increase over time, and we did not find significant association between C-section and early childhood diseases. High C-section rate has a greater impact on maternal and child health as well as the burden on the health care system. We recommend raising public awareness of the negative impact of unnecessary C-section in Bangladesh.

## Introduction

Cesarean delivery (C-section) is a surgical procedure that is often performed or recommended when the life of the mother or child is at risk [1]. Recently, it has become a preferred choice as a mode of delivery among women because they believed that it is painless, comfortable, safer, and healthier than normal delivery [2]. This choice may have increased unnecessary C-section and could harm the mother and child health [3].

The prevalence of the C-section is expeditiously growing in many developed and developing countries [4, 5]. During the last decades, unnecessary C-section has increased rapidly [6]. It is rising significantly, as more than half of the women willingly undergo C–section [7]. A trend analysis based on data from 121 countries reported that, from 1990 to 2014, the average C-section rates increased by 12.4%, and it annually increased by 4.4% [8]. Moreover, a 2004–2008 World Health Organization (WHO) survey documented an average global rate of C-section was 25.7%, and the rate was 27.3% in Asia, 29.2% in Latin America, and 19.0% in Europe [8, 9]. In Bangladesh, the rate increased six times from 3.5% in 2004 to 23% in 2014 [10].

There are several risks associated with C-section for mothers and children [11, 12]. Babies born in C-section are at risk of developing asthma, obesity, type 1 diabetes, allergic diseases [11, 12], Crohn's disease [13], and so on. Moreover, C-section babies may develop neurodevelopmental disorders, such as attention deficit hyperactivity disorder, autism spectrum disorder, learning disabilities, etc. [14–18].

In Bangladesh, young children, in general, are suffering from several common diseases such as fever, cough, short/ difficulty in breathing, diarrhea etc. [19]. Several studies investigated the impact of socio-demographic, maternal, or child characteristics on specific childhood diseases [16, 20–22]. For example, Imran et al. [22] investigated the potential risk factors for early childhood acute respiratory infections; Pathelaet et al. [16] studied the risk factors for the diarrheal disease of young children in Bangladesh. However, to the best of our knowledge, there is no published research on the association between C-section and early childhood diseases and/or identify potential risk factors that may influence the overall common childhood disease in Bangladesh. Therefore, our main objective was to study the association between C-section and common childhood diseases and to identify potential factors that may influence childhood diseases.

## Materials and methods

### Data source and study design

We used two different survey data sets of 2011, 2014 Bangladesh Demographic and Health Survey (BDHS) and 2012, 2019 Multiple Indicator Cluster Survey (MICS). The BDHS is a large household survey produced by the Demographic and Health Surveys Program, and the MICS is also a large, multi-dimensional household survey conducted by UNICEF. Both surveys collects maternal and child health indicators. Details of the methodology and sampling procedure of both surveys were published elsewhere [23, 24]. We included women who gave birth three years prior to the survey. Children who died or did not live with their mother or who were over 3 years of age at the time of the survey were excluded from the analysis. The final analysis included 4748, 4527, 7248, and 8747 mother-child pairs from 2011 BDHS, 2014 BDHS, 2012 MICS, and 2019 MICS, respectively.

### Outcome variable

For creating the outcome variable (childhood disease), we used several variables such as fever, short/ rapid breaths, cough, blood in stools, and diarrhea in the two weeks before or during the survey. We created a count variable that means the frequency of the diseases of the children. Here, the number of diseases for a child varies from 0 to 5. The zero means the child did not suffer any above-mentioned diseases in the two weeks before or during the survey.

### Exposure variable

The exposure variable was the type of delivery (C-section vs. normal delivery), which is a binary variable.

### Potential confounding variables

We considered important confounding variables and/or covariates are mothers age, place of residence, division, mother’s education, wealth index, religion, mother’s body mass index (BMI), breastfeeding status, child age, child sex, and child’s size at birth.

### Statistical analyses

Descriptive statistics of each of the selected covariates and distribution of type of delivery were shown by adjusting the sampling weight of the survey. Similarly, weighted percentages were calculated to compare demographic and socioeconomic characteristics among the type of delivery. Pearson's chi-squared test was used to determine the association between C-section (vs. normal delivery) and other covariates. As our outcome is a count variable, frequency of diseases, we first applied Poisson regression models. However, due to over- dispersion in the data, we then applied negative binomial (NB) regression models. We first fitted univariate models to estimate the effect of C‐section on the outcome variable (disease count). Subsequently, we also fitted univariate models using all potential covariates. We used an arbitrary p—value of ≤ 0.20 as a criterion to include covariates in the multivariable models. We used stepwise procedures to select the best model. Therefore, in our final model, we had included all significant covariates and some key variables related to the outcome. To account for the complex survey design, we used the *Svyset* command in Stata (StataCorp LP, College Station, Texas). The *Svyset* command helps us to use design elements such as the primary sampling unit, strata, cluster, and sample weight.

#### Ethics approval

Our study was exempt from the ethical review approval because we used publicly available de-identified data.

## Results

**Fig 1:**
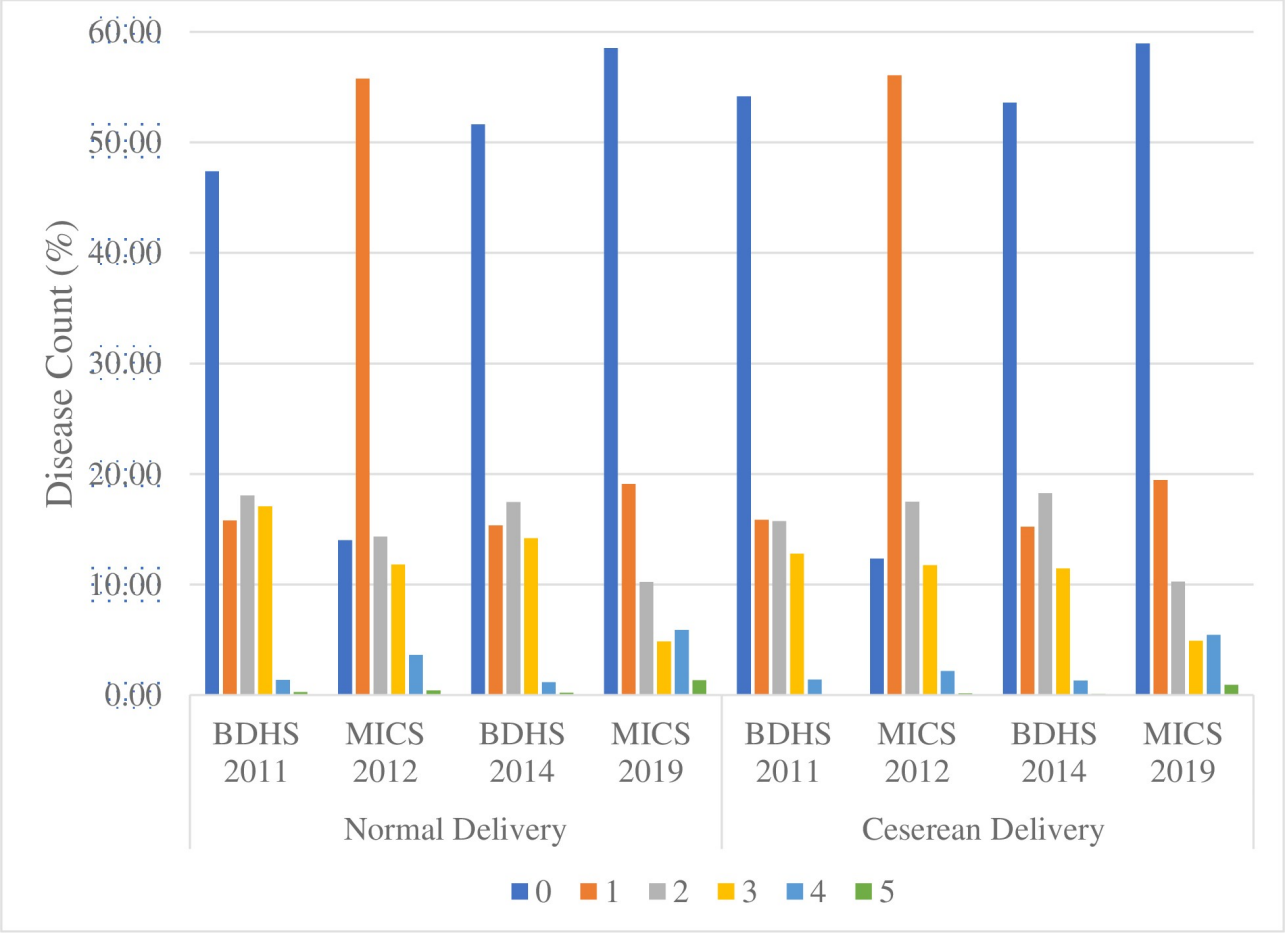
Distribution of common childhood disease by delivery type and survey years.

The prevalence of C-section increased over time in both surveys. In BDHS, the rate increased from 17.95% in 2011 to 23.33% in 2014. Also, in MICS, the prevalence almost doubled over an eight-year period (17.74% in 2012 to 35.41% in 2019). Other than 2012 MICS, the distribution of common childhood diseases across survey years were fairly similar. More than half (48 to 52%) of the children had no diseases, followed by 15 to 19% had one disease in two weeks prior to the survey. The proportion of the disease counts by delivery type was approximately similar across survey years and between surveys (**Fig 1**).

**Tables 1** and **2** outlines the maternal and child characteristics between C-section and normal delivery for BDHS and MICS surveys, respectively. The distribution of C-section by mother’s age increased over time in both surveys. For example, in BDHS, the rate of C-section among 25–29 year old mothers increased from 19.8% in 2011 to 26.53% in 2014; similarly in MICS the rate doubled (17.73% vs. 36.68%). In both surveys, women from Dhaka division, living in urban areas with higher education and higher wealth status, had a higher proportion of having C-section in recent surveys compared to the previous surveys. Among the child's characteristics, the baby's size at birth was one of the significant factors found to be associated with C-section. For example, one-third of the baby's size larger than average was delivered by cesarean section in 2014 BDHS.

**Table 1:** Sample characteristics of mother and children by delivery status, BDHS 2011–2014.

	2011 BDHS			2014 BDHS		
	Normal Delivery	Caesarean Delivery	p-values	Normal Delivery	Caesarean Delivery	p-values
	n (%)	n (%)		n (%)	n (%)	
Mothers age			<0.001			0.01
< 15–19	789 (86.8)	120 (13.2)		739 (79.55)	190 (20.45)	
20–24	1439 (81.76)	321 (18.24)		1218 (77.93)	345 (22.07)	
25–29	964 (80.2)	238 (19.8)		853 (73.47)	308 (26.53)	
30–34	459 (78.73)	124 (21.27)		461 (76.2)	144 (23.8)	
35+	229 (82.67)	48 (17.33)		199 (74.25)	69 (25.75)	
Place of Residence			<0.001			<0.001
Urban	1070 (72.1)	414 (27.9)		917 (63.5)	527 (36.5)	
Rural	2821 (86.59)	437 (13.41)		2553 (82.84)	529 (17.16)	
Division			<0.001			<0.001
Dhaka	431 (83.53)	85 (16.47)		540 (67.67)	258 (32.33)	
Barishal	843 (85.15)	147 (14.85)		432 (80.6)	104 (19.4)	
Chittagong	604 (78.85)	162 (21.15)		713 (80.84)	169 (19.16)	
Khulna	408 (73.12)	150 (26.88)		345 (65.59)	181 (34.41)	
Rajshahi	471 (79.7)	120 (20.3)		403 (73.14)	148 (26.86)	
Rangpur	507 (86.67)	78 (13.33)		435 (80.11)	108 (19.89)	
Sylhet	627 (85.19)	109 (14.81)		602 (87.25)	88 (12.75)	
Mothers Education			<0.001			<0.001
No-education	765 (95.86)	33 (4.14)		567 (92.95)	43 (7.05)	
Primary	1264 (91.26)	121 (8.74)		1103 (88.52)	143 (11.48)	
Secondary	1700 (79.4)	441 (20.6)		1570 (73.36)	570 (26.64)	
Higher	162 (38.76)	256 (61.24)		230 (43.4)	300 (56.6)	
Wealth Index			<0.001			<0.001
Poorest	990 (97.06)	30 (2.94)		908 (94.58)	52 (5.42)	
Poorer	825 (91.46)	77 (8.54)		759 (88.46)	99 (11.54)	
Middle	777 (86.24)	124 (13.76)		707 (81.17)	164 (18.83)	
Richer	750 (79.2)	197 (20.8)		662 (70.8)	273 (29.2)	
Richest	549 (56.48)	423 (43.52)		434 (48.12)	468 (51.88)	
Religion			0.02			0.35
Non-Muslim	368 (76.83)	111 (23.17)		259 (72.14)	100 (27.86)	
Muslim	3523 (82.64)	740 (17.36)		3211 (77.06)	956 (22.94)	
Mothers BMI			<0.001			<0.001
Underweight	1248 (90.37)	133 (9.63)		976 (87.46)	140 (12.54)	
Normal weight	1917 (84.71)	346 (15.29)		1684 (80.73)	402 (19.27)	
Overweight	545 (69.78)	236 (30.22)		629 (64.65)	344 (35.35)	
Obese	181 (57.1)	136 (42.9)		181 (51.57)	170 (48.43)	
Breastfeeding			0.006			0.071
No	482 (78.12)	135 (21.88)		469 (73.86)	166 (26.14)	
Yes	3409 (82.64)	716 (17.36)		3001 (77.13)	890 (22.87)	
Childs Sex			0.059			0.036
Male	1937 (81.01)	454 (18.99)		1755 (75.39)	573 (24.61)	
Female	1954 (83.11)	397 (16.89)		1715 (78.03)	483 (21.97)	
Child's age, years			0.030			0.005
0	1337 (80.49)	324 (19.51)		1084 (74.5)	371 (25.5)	
1	1258 (81.74)	281 (18.26)		1173 (76.02)	370 (23.98)	
2	1296 (84.05)	246 (15.95)		1213 (79.38)	315 (20.62)	
Childs size at Birth			0.006			<0.001
Average	2659 (82.68)	557 (17.32)		2362 (76.99)	706 (23.01)	
Smaller than average	717 (83.08)	146 (16.92)		704 (80.27)	173 (19.73)	
Larger than average	515 (77.68)	148 (22.32)		403 (69.48)	177 (30.52)	

Numbers in the parenthesis indicates row percentages.

**Table 2:** Sample characteristics of mother and children by delivery status, MICS 2012 and 2019.

	2012 MICS			2019 MICS		
	Normal Delivery	Caesarean Delivery	p-values	Normal Delivery	Caesarean Delivery	p-values
	n (%)	n (%)		n (%)	n (%)	
Mothers age			0.012			<0.001
< 15–19	730 (82.3)	157 (17.7)		803 (66.36)	407 (33.64)	
20–24	2088 (81.25)	482 (18.75)		1849 (62.83)	1094 (37.17)	
25–29	1810 (82.27)	390 (17.73)		1552 (63.32)	899 (36.68)	
30–34	819 (81.74)	183 (18.26)		951 (64.39)	526 (35.61)	
35+	515 (87.44)	74 (12.56)		495 (74.32)	171 (25.68)	
Place of Residence			<0.001			<0.001
Urban	851 (70.51)	356 (29.49)		886 (52.55)	800 (47.45)	
Rural	5111 (84.61)	930 (15.39)		4764 (67.47)	2297 (32.53)	
Division			<0.001			<0.001
Dhaka	1421 (77.52)	412 (22.48)		1336 (59.59)	906 (40.41)	
Barishal	591 (89.14)	72 (10.86)		572 (72.41)	218 (27.59)	
Chittagong	1339 (88.5)	174 (11.5)		1331 (73.9)	470 (26.1)	
Khulna	679 (69.5)	298 (30.5)		568 (46.67)	649 (53.33)	
Rajshahi	504 (77.54)	146 (22.46)		513 (58.7)	361 (41.3)	
Rangpur	806 (88.47)	105 (11.53)		722 (67.54)	347 (32.46)	
Sylhet	622 (88.73)	79 (11.27)		608 (80.64)	146 (19.36)	
Mothers Education			<0.001			<0.001
No-education	1306 (94.98)	69 (5.02)		634 (87.81)	88 (12.19)	
Primary	1929 (91.25)	185 (8.75)		1593 (81.19)	369 (18.81)	
Secondary	2199 (78.65)	597 (21.35)		2801 (62.7)	1666 (37.3)	
Higher	528 (54.83)	435 (45.17)		622 (38.97)	974 (61.03)	
Wealth Index			<0.001			<0.001
Poorest	1857 (94.89)	100 (5.11)		1759 (86.14)	283 (13.86)	
Poorer	1404 (90.87)	141 (9.13)		1319 (74.06)	462 (25.94)	
Middle	1166 (86.24)	186 (13.76)		1041 (62.79)	617 (37.21)	
Richer	917 (73.48)	331 (26.52)		926 (54.28)	780 (45.72)	
Richest	618 (53.93)	528 (46.07)		605 (38.78)	955 (61.22)	
Religion			0.237			0.009
Non-Muslim	646 (80.75)	154 (19.25)		503 (60.46)	329 (39.54)	
Muslim	5316 (82.44)	1132 (17.56)		5147 (65.03)	2768 (34.97)	
Breastfeeding						
No	5909 (82.36)	1266 (17.64)		5632 (64.63)	3082 (35.37)	
Yes	36 (66.67)	18 (33.33)		18 (54.55)	15 (45.45)	
Childs Sex						
Male	2971 (80.95)	699 (19.05)		2828 (63.1)	1654 (36.9)	
Female	2991 (83.59)	587 (16.41)	0.003	2822 (66.17)	1443 (33.83)	0.227
Child's age, years						
0	2777 (81.25)	641 (18.75)		2602 (62.61)	1554 (37.39)	
1	2864 (82.49)	608 (17.51)	0.003	2681 (65.87)	1389 (34.13)	0.003
2	321 (89.66)	37 (10.34)		367 (70.44)	154 (29.56)	
Childs size at Birth						
Average	3656 (82.83)	758 (17.17)	<0.001	1033 (66.26)	526 (33.74)	<0.001
Smaller than average	1303 (84.72)	235 (15.28)		4033 (66.28)	2052 (33.72)	
Larger than average	698 (71.96)	272 (28.04)		527 (51.07)	505 (48.93)	

Numbers in the parenthesis indicates row percentages.

**Tables 3** and **4** show the results of the multivariable negative binomial regression models for BDHS and MICS surveys estimating the effects of C-section (vs. normal delivery) on childhood diseases after adjusting for maternal and child’s characteristics. No statistically significant effects of C-section on childhood diseases were observed in both sets of surveys. However, having a C-section appears to increase the risk of childhood disease by 5% (RR: 1.05, 95% CI: 0.95, 1.17, p = 0.33) only in 2014 BDHS. Overall, the risk of common childhood diseases differed significantly in all survey years by division, child's age, and child’s size at birth. In both surveys, children living in Chittagong division had a higher risk [(2011 BDHS RR: 1.22, 95% CI: 1.08, 1.38) and (2019 MICS RR:1.21, 95% CI: 1.08, 1.35)] of having disease compared to Dhaka division. The risk of having common childhood diseases decreases as children grow. For example, one year old child had a 42% (RR: 1.42, 95% CI: 1.19, 1.71, p <0.001) and 19% (RR: 1.19, 19% CI: 1.08, 1.31, p = 0.001) higher risk of having common childhood diseases in 2019 MICS and 2014 BDHS, respectively. There were significant differences in common childhood diseases among children born with smaller and larger than average sizes. Children born with either smaller or larger than average size had a higher likelihood of having common childhood diseases. Mothers' age plays a key role in childhood disease, compared with younger mothers (15–19 years) children born to young adult mothers had a lower risk of having childhood diseases was observed in 2019 MICS, not other surveys. For example, children born to 30–34 years old mothers had 18% less risk of having childhood disease. There was a significant increase in childhood disease among the children who were born to mothers with lower levels of education. Similarly, children born to a lower socio-economic status family had a higher risk of having common childhood diseases.

**Table 3:** Factors associated with cesarean vs normal delivery and common childhood diseases, BDHS 2011and 2014.

	2011 BDHS		2014 BDHS	
	IRR (95% CI)	p-value	IRR (95% CI)	p-value
Cesarean delivery				
No	Reference		Reference	
Yes	0.92 (0.82, 1.02)	0.129	1.05 (0.95, 1.17)	0.33
Mothers age				
15–19	Reference		Reference	
20–24	1.07 (0.97, 1.18)	0.195	1.02 (0.88, 1.19)	0.751
25–29	0.97 (0.87, 1.09)	0.599	0.94 (0.83, 1.07)	0.356
30–34	0.99 (0.86, 1.13)	0.826	0.95 (0.80, 1.12)	0.532
35+	1.18 (0.99, 1.41)	0.064	0.84 (0.70, 1.01)	0.064
Division				
Dhaka	Reference		Reference	
Barishal	1.05 (0.91, 1.22)	0.485	1.11 (0.93, 1.32)	0.232
Chittagong	1.22 (1.08, 1.38)	0.001	1.19 (1.04, 1.36)	0.011
Khulna	1.11 (0.95, 1.29)	0.177	0.97 (0.82, 1.14)	0.704
Rajshahi	1.05 (0.92, 1.19)	0.50	1.08 (0.92, 1.27)	0.328
Rangpur	1.06 (0.9, 1.25)	0.465	1.07 (0.89, 1.28)	0.474
Sylhet	1.11 (0.98, 1.25)	0.092	1.14 (0.99, 1.31)	0.07
Education				
Higher	Reference		Reference	
Secondary	1.14 (0.97, 1.35)	0.112	1.04 (0.88, 1.22)	0.65
Primary	1.26 (1.05, 1.51)	0.012	1.13 (0.94, 1.35)	0.185
No-education	1.09 (0.89, 1.35)	0.393	1.16 (0.96, 1.41)	0.125
Wealth index				
Richest	Reference		Reference	
Richer	1.07 (0.94, 1.22)	0.305	1.09 (0.94, 1.25)	0.245
Middle	1.06 (0.93, 1.2)	0.371	1.16 (0.99, 1.35)	0.064
Poorer	1.03 (0.9, 1.19)	0.647	1.08 (0.90, 1.28)	0.407
Poorest	1.29 (1.13, 1.48)	<0.001	1.10 (0.92, 1.32)	0.305
Child's sex				
Female	Reference		Reference	
Male	1.11 (1.03, 1.19)	0.007	1.05 (0.97, 1.14)	0.254
Child's age, years				
2	Reference		Reference	
1	1.18 (1.07, 1.29)	0.001	1.15 (1.04, 1.26)	0.006
0	1.19 (1.08, 1.30)	<0.001	1.19 (1.08, 1.31)	0.001
Child's size at birth				
Average	Reference		Reference	
Smaller than average	1.23 (1.12, 1.35)	<0.001	1.13 (1.02, 1.25)	0.021
Larger than average	1.23 (1.13, 1.35)	<0.001	1.00 (0.86, 1.15)	0.961

**Table 4:** Factors associated with cesarean vs normal delivery and common childhood diseases, MICS 2012–2019.

	2012 MICS		2019 MICS	
	IRR (95% CI)	p-value	IRR (95% CI)	p-value
Cesarean delivery				
No	Reference		Reference	
Yes	0.98 (0.92, 1.04)	0.492	0.95 (0.88, 1.03)	0.209
Mothers age				
15–19	Reference		Reference	
20–24	0.98 (0.91, 1.05)	0.511	0.92 (0.82, 1.03)	0.146
25–29	0.98 (0.91, 1.05)	0.549	0.95 (0.84, 1.06)	0.353
30–34	0.97 (0.89, 1.05)	0.458	0.82 (0.72, 0.94)	0.005
35+	0.95 (0.86, 1.05)	0.307	0.71 (0.59, 0.84)	<0.001
Division				
Dhaka	Reference		Reference	
Barishal	1.12 (1.03, 1.22)	0.006	1.17 (1.01, 1.34)	0.036
Chittagong	0.93 (0.87, 1.00)	0.053	1.21 (1.08, 1.35)	0.001
Khulna	1.16 (1.09, 1.24)	<0.001	1.15 (1.02, 1.29)	0.018
Rajshahi	1.14 (1.07, 1.22)	<0.001	1.18 (1.04, 1.33)	0.008
Rangpur	1.05 (0.98, 1.12)	0.176	1.07 (0.94, 1.23)	0.311
Sylhet	1.01 (0.93, 1.09)	0.85	0.6 (0.5, 0.72)	<0.001
Education				
Higher	Reference		Reference	
Secondary	1.07 (0.99, 1.15)	0.099	0.99 (0.89, 1.1)	0.835
Primary	1.04 (0.96, 1.13)	0.329	1.05 (0.92, 1.19)	0.499
No-education	1.07 (0.97, 1.17)	0.163	0.84 (0.69, 1.01)	0.068
Wealth Index				
Richest	Reference		Reference	
Richer	0.97 (0.89, 1.04)	0.381	0.96 (0.84, 1.09)	0.501
Middle	1.01 (0.93, 1.1)	0.765	0.99 (0.88, 1.12)	0.919
Poorer	1.01 (0.93, 1.11)	0.749	1.17 (1.02, 1.33)	0.023
Poorest	1.04 (0.96, 1.14)	0.331	1.08 (0.94, 1.24)	0.266
Child's sex				
Female	Reference		Reference	
Male	1.00 (0.95, 1.04)	0.83	1.12 (1.04, 1.19)	0.002
Child's age, years				
2	Reference		Reference	
1	1.19 (1.07, 1.31)	0.001	1.42 (1.19, 1.71)	<0.001
0	1.12 (1.02, 1.24)	0.023	1.40 (1.17, 1.68)	<0.001
Child's size at birth				
Average	Reference		Reference	
Smaller than average	1.07 (1.01, 1.13)	0.023	1.25 (1.14, 1.37)	<0.001
Larger than average	1.01 (0.95, 1.08)	0.717	1.22 (1.10, 1.36)	<0.001

## Discussion

In this study, we investigated the relationship between C-section (vs. normal delivery) and early childhood diseases in Bangladesh using multiple nationally representative survey datasets. We also investigated the factors associated with common childhood diseases. We observed that for BDHS (2011), MICS (2012), BDHS (2014), and MICS (2019), the prevalence of cesarean deliveries was 17.95%, 17.74%, 23.3%, and 35.41%, respectively. The distributions of childhood diseases were approximately similar in both cesarean and normal delivery in all survey datasets across the survey years.

In multivariable negative binomial regression models, there was no significant association between C-section and common childhood diseases. Similar results have been observed by Gondwe et al. in a similar population setting in India [25, 26]. However, there are other studies in both developed and developing countries have found that C-section is significantly associated with childhood diseases (e.g. asthma and respiratory diseases). Moreover, among the key factors, division -geographical locations, age of the child, and child’s size at birth had a significant impact on the childhood disease in all surveys. Maternal age, education, wealth status, have also been found to be significant in some survey years. We also observed other factors such as division, child's age, and size at birth were significantly associated with childhood disease in all surveys.

We have noticed that the delivery rate for the C-section was higher particularly in the Dhaka division compared with other geographical divisions in Bangladesh. An earlier study found that women in the division of Chittagong, Dhaka, Khulna and Rajshahi were more likely to go C-section [27]. For instance, the risk of disease was higher in the Khulna division in the MICS surveys. Most of the women in these areas are educated and they belong to middle-class and rich families and have access to and ability to undergo C-section delivery [28]. Nowadays, educated pregnant women want to avoid vaginal delivery in fear of labor pain and other conveniences. Perhaps these are the most important reasons for the increased rate of cesarean delivery in Bangladesh.

Our study findings also confirmed that the highest rate of C-section has occurred among secondary or higher educated females. Since education is directly linked to women's autonomy, they are more economically solvent and mostly living in urban areas, can decide to give birth through the C-section. However, some studies show that women's choice of C-Section has no visible link with their educational level [29, 30]. In terms of wealth, health facilities were higher for the rich families than for the mid- and poorer families. In comparison with the poorest or poorest families, the rates for C-section were higher among the rich families [31]. This might be due to financial issues since the wealthy family can pay C-section costs.

The analyses of this study confirmed that childhood disease is associated with maternal age, according to MICS data. An earlier study showed that children born to younger teenage mothers were found to have a relatively high risk of diarrhea, cough, and fever [32]. This is due to the fact that maternal age is linked with some adverse pregnancy outcomes and a higher risk of developing medical conditions such as hypertension, diabetes, or other causes. However, in the BDHS data, we did not observe any significant relationship between the ages of the mothers and the risk of short-term diseases.

Our study has several strengths: first, to our knowledge, this the first study to examine delivery-section and childhood diseases in Bangladesh; second, we used the latest available four data sets from two nationally representative surveys, third, we used proper data analyses methodology in which we accounted for all complex survey designs. However, there are some limitations of the study: first, we used cross-sectional survey data and the childhood disease changes over time and the reported association may change in the longitudinal studies, although our study exposure variable, C-section, was a time-independent variable; second, an important maternal factor complications during pregnancy that have a significant number of missing values and could not consider them in the analyses; third, data on reasons for C-section were not available to capture an understand of the choice.

## Conclusions

Our study shows that cesarean delivery in Bangladesh has continued to increase rapidly over time, and we did not find any significant association between cesarean delivery and early childhood diseases. The study also confirmed that childhood disease is significantly related to maternal age, geographical division, maternal education and wealth index, age of the child, and birth size.
